# Nerve Stretching in Inveterate Trigeminal Neuralgia

**Published:** 1892-09

**Authors:** 


### Nerve-Stretching in Inveterate Trigeminal Neuralgia.

Dr. James Stewart (*Montreal Medical Journal*) says: Nerve-stretching gives either complete or great relief in the majority of cases. Relief is not permanent in more than 5 per cent. of cases. If the pain should return the operation should be repeated, even several times, before resorting to a neurectomy, or ligature of the common carotic. If the pain is not strictly and always limited to one branch of the nerve, several branches should be stretched. As relief does not always immediately follow the stretching a second operation should not be undertaken until some time has elapsed.

